# Characterization of the SUMO-Binding Activity of the Myeloproliferative and Mental Retardation (MYM)-Type Zinc Fingers in ZNF261 and ZNF198

**DOI:** 10.1371/journal.pone.0105271

**Published:** 2014-08-18

**Authors:** Catherine M. Guzzo, Alison Ringel, Eric Cox, Ijeoma Uzoma, Heng Zhu, Seth Blackshaw, Cynthia Wolberger, Michael J. Matunis

**Affiliations:** 1 Department of Biochemistry and Molecular Biology, Bloomberg School of Public Health, Johns Hopkins University, Baltimore, Maryland, United States of America; 2 Department of Biophysics and Biophysical Chemistry, Johns Hopkins University School of Medicine, Baltimore, Maryland, United States of America; 3 The Solomon H. Snyder Department of Neuroscience, Johns Hopkins University School of Medicine, Baltimore, Maryland, United States of America; 4 Department of Pharmacology and Molecular Sciences, Johns Hopkins University, Baltimore, Maryland, United States of America; University of Minnesota, United States of America

## Abstract

SUMO-binding proteins interact with SUMO modified proteins to mediate a wide range of functional consequences. Here, we report the identification of a new SUMO-binding protein, ZNF261. Four human proteins including ZNF261, ZNF198, ZNF262, and ZNF258 contain a stretch of tandem zinc fingers called myeloproliferative and mental retardation (MYM)-type zinc fingers. We demonstrated that MYM-type zinc fingers from ZNF261 and ZNF198 are necessary and sufficient for SUMO-binding and that individual MYM-type zinc fingers function as SUMO-interacting motifs (SIMs). Our binding studies revealed that the MYM-type zinc fingers from ZNF261 and ZNF198 interact with the same surface on SUMO-2 recognized by the archetypal consensus SIM. We also present evidence that MYM-type zinc fingers in ZNF261 contain zinc, but that zinc is not required for SUMO-binding. Immunofluorescence microscopy studies using truncated fragments of ZNF198 revealed that MYM-type zinc fingers of ZNF198 are necessary for localization to PML-nuclear bodies (PML-NBs). In summary, our studies have identified and characterized the SUMO-binding activity of the MYM-type zinc fingers in ZNF261 and ZNF198.

## Introduction

Small Ubiquitin-related Modifiers (SUMOs) are reversible post-translational protein modifications that are conjugated to lysine residues on substrate proteins by an enzymatic cascade. Interactions between SUMO-modified proteins and SUMO-binding proteins containing SUMO-interacting motifs (SIMs) regulate a wide range of functional consequences including formation of multi-protein complexes and changes in protein localization, activity, solubility, and stability [1], [2]. The functional diversity of the SUMO signal is due, in part, to expression of three SUMO paralogs and formation of polymeric SUMO. Mammals contain three isoforms of SUMO known as SUMO-1, SUMO-2 and SUMO-3. SUMO-1 is ∼50% identical to SUMO-2 and SUMO-3. SUMO-2 and SUMO-3 are frequently referred to as SUMO-2/3 because they are ∼96% identical and may be functionally redundant. SUMO-2/3 contains a consensus SUMOylation motif, ΨKXE/D, and can therefore readily form polymeric chains.

The archetypal consensus SIM consists of four hydrophobic amino acids adjacent to a cluster of negatively charged residues [3]-[8]. Structural studies revealed that this SIM forms an extended β-strand that inserts between the α-helix and second β-strand in all three SUMO paralogs [4], [7]–[10]. However, little is understood regarding how proteins preferentially recognize SUMO-1 or SUMO-2, although for some SIM containing proteins the cluster of negatively charged residues confers binding specificity for SUMO-1 over SUMO-2 [4], [11].

To address SUMO paralog-selectivity, we initiated high-throughput studies using protein microarrays to identify novel SUMO-1, SUMO-2, and SUMO-2 polymeric chain binding proteins. Among the identified proteins was ZNF261, which contains a stretch of unique tandem zinc fingers called MYM-type zinc fingers (MYeloproliferative and Mental retardation-type zinc fingers). Tandem MYM-type zinc fingers are found in three other human proteins including ZNF198, ZNF262, and ZNF258 [12]. Additionally, ZNF237 and ZMYM1 genes encoding for MYM-type zinc finger containing proteins have been sequenced [13], [14]. However, the major splice variant of ZNF237 produces a protein containing only a single MYM-type zinc finger and current evidence for ZMYM1 is only available at the transcript level. The function of MYM-type zinc finger containing proteins are not fully understood, however, proteins within this family have been linked to myeloproliferative syndromes [15], [16], mental retardation [17], transcriptional regulation [18], [19], DNA repair [20], and telomere maintenance [21].

ZNF198 is the best studied member of the MYM-type zinc finger family of proteins. Translocation of the ZNF198 and Fibroblast Growth Factor Receptor 1 (FGFR1) genes has been linked to atypical myeloproliferative disease [15], [16]. Intriguingly, several studies have previously revealed connections between ZNF198 and sumoylation. First, studies have shown that ZNF198 is modified by SUMO-1 and that this modification is required for its localization to PML nuclear bodies [22]. Second, studies have shown that ZNF198 affects gene expression in part through a mechanism that involves interactions with SUMO-modified HDAC1 [18]. Although direct interactions between ZNF198 and SUMO were not demonstrated, a fragment of ZNF198 containing the MYM-type zinc-finger domain was found to be necessary and sufficient for binding specifically to SUMO-2 modified, but not unmodified, HDAC1.

In this report, we characterized ZNF261 as a newly identified SUMO-binding protein. Our analysis revealed that ZNF261 and ZNF198 interact with SUMO through the MYM-type zinc fingers rather than a consensus SIM. We also provide evidence that the MYM-type zinc fingers in ZNF198 are important for localization to PML-nuclear bodies (PML-NBs). Taken together, our findings establish that the MYM-type zinc fingers are legitimate SUMO-binding domains.

## Materials and Methods

### Antibodies

Anti-GST (B-14, sc-138, Santa Cruz Biotechnology), anti-myc (9E10, sc-40, Santa Cruz Biotechnology), anti-PML (A301-167A, Bethyl) were used.

### Cell culture

The U2OS cell line used in this study is an established cell line that is commercially available from American Type Culture Collection (ATCC, U2OS, HTB-96). U2OS cells were grown in Dulbecco's modified Eagle's medium supplemented with 10% fetal bovine serum and 10 mM Hepes. Cells were cultured at 37°C in a humidified incubator with 5% CO_2_.

### CD measurements

CD measurements of ZNF261 samples (75 µM) were performed on a Jasco J-810 spectropolarimeter using a 0.2 mm pathlength cuvette (Jasco). Protein absorbance was measured in the UV range between 260 and 190 nm. Samples were scanned at 100 nm/min and 10 scans were collected per sample. High-tension voltage was kept below 500 V during all scans. Treated samples were dialyzed overnight against 50 mM EDTA, 100 mM sodium acetate pH 5.5 or 6 M GuHCl.

### Equilibrium analytical ultracentrifugation

Equilibrium analytical ultracentrifugation experiments were performed in 20 mM HEPES, pH 7.6, 350 mM NaCl, 5 mM DTT, and 10 µM ZnCl_2_ at 10°C in an An60-Ti rotor on a Beckman Optima XL-I. Recombinant MYM SIMs from ZNF261 (amino acids 236–490) were run in the analytical ultracentrifuge at three different concentrations (10, 20, and 40 µM) at rotor speeds of 15,000, 20,000, and 25,000 rpm. Three different concentrations of SUMO-2(x2) (20, 40, 80 µM) were added to 20 µM MYM SIMs from ZNF261 and run in the analytical ultracentrifuge using the same protocol. Approach to equilibrium was monitored using SEDFIT [23] and sedimentation equilibrium absorbance data was collected at 280 nm every two hours for 10–16 hours at each rotor speed. Only scans attaining equilibrium were used for data analysis, which were globally fit to an A+B → AB binding model using SEDPHAT [24]. Confidence intervals were calculated using F-statistics and the error surface projection method used in SEDPHAT [24]. The partial specific volume for ZNF261 (0.7122 ml/g) and the SUMO-2(x2) (0.7190 ml/g) were calculated using SedNTerp [25]. For each sample, total protein concentration did not exceed 2 mg/ml and absorbance values were under 1.0.

### Immunofluorescence microscopy

Cells grown on coverslips were washed with phosphate-buffered saline (PBS) and then fixed and permeabilized at room temperature with PBS containing 2% formaldehyde/0.2% Triton X-100. After 10 min, cells were washed with PBS and fixed with 2% formaldehyde in PBS for 20 min at room temperature. Cells were washed with PBS and incubated with the indicated primary antibodies for 1 hour. Cells were washed and incubated with Alexa Fluor–conjugated goat anti-rabbit immunoglobulinG (IgG) or Alexa Fluor–conjugated goat anti-mouse IgG (Invitrogen) for 1 hour. Coverslips were washed with PBS and mounted with mounting solution containing 100 mM tris (pH 8.8), 50% glycerol, 2.5% DABCO (Sigma), and 4′,6′-diamidino-2-phenylindole (0.2 mg/ml). Images were obtained on a Zeiss Observer.Z1 microscope.

### Immunoprecipitation

U2OS cells were transfected, incubated for 24 hours, and lysed in buffer A (50 mM Tris-HCl pH 8.0, 2 mM EDTA, 150 mM NaCl, and 1% NP-40) containing 1 mM PMSF, 0.3 µM aprotinin, 10 µM leupeptin, 1 µM pepstatin A, 0.025 U/µl benzonase, and 10 mM N-ethyl-maleimide (Sigma). Cell lysates were sonicated on ice and centrifuged at 14,000 rpm and 4°C for 10 min. Soluble cell lysates and anti-Flag-agarose beads (A2220, Sigma) or anti-myc-agarose beads (sc-40 AC, Santa Cruz Biotechnology) were incubated for 3 hours and washed five times with buffer A. Bound proteins were eluted with SDS-PAGE loading buffer.

### In vitro binding assays

Recombinant GST, GST-tagged SUMO-1, SUMO-2, SUMO-2(QFI), or SUMO-2(x3) (8 µg protein) were diluted into 100 µl of 1X PBS, 0.05% Tween 20 and incubated in glutathione-coated 96-well plates (Pierce Biotechnology). Following overnight incubation at 4°C, wells were blocked for 1 h at room temperature with 2% bovine serum albumin in assay buffer (20 mM HEPES-KOH pH 7.3, 110 mM potassium acetate, 2 mM magnesium acetate, 1 mM EGTA, 0.05% Tween 20). ZNF261 and ZNF198 proteins were produced by *in vitro* transcription and translation in rabbit reticulocyte lysate in the presence of [^35^S]methionine according to manufacturer's instructions (Promega). *In vitro* translated proteins (10 µl) were diluted into 100 µl of assay buffer and incubated with the immobilized proteins for 1 h at room temperature. Where indicated binding reactions were performed in the presence of 50 mM EDTA, 100 mM sodium acetate pH 5.5, or 10 mM 1,10-phenanthroline, or 10 mM 1,7-phenanthroline. Unbound proteins were removed by washing and bound proteins were eluted with SDS sample buffer and resolved by SDS-PAGE and autoradiography. Equal loading of immobilized proteins was verified by anti-GST immunoblot after binding. Relative binding was determined by loading equal cpm of radiolabeled protein per well followed by liquid scintillation detection of eluted proteins. All binding assays were repeated in triplicate.

Binding assays using recombinant His-tagged ZNF261 (amino acids 1-495) proteins were performed following overnight dialysis against assay buffer, or 50 mM EDTA, 100 mM sodium acetate pH 5.5, or 1,10-phenanthroline, or 1,7-phenanthroline. Binding assays were performed as described above and bound proteins were eluted with SDS sample buffer and detected by anti-His immunoblot.

### Plasmids

ZNF261 (amino acids 1-495) entry clone was obtained from the Ultimate Human ORF collection (Invitrogen). To generate a clone for protein expression in bacteria or rabbit reticulocyte lysate, recombination reactions were performed between the entry clone and Gateway pDEST17 Vector according to manufacturer's instructions (Invitrogen). Amino acid substitutions in ZNF261 were generated by PCR-based QuikChange site-directed mutagenesis (Stratagene) using the wild-type plasmid. ZNF261 and ZNF198 fragments were generated by PCR, digested with appropriate restriction enzymes, and ligated into pcDNA6/myc-His A (Invitrogen). All mutations and constructs were verified by DNA sequence analysis. Full-length ZNF198 cDNA was a gift from Hongtao Yu (The University of Texas Southwestern Medical Center, Dallas, TX). The construct for expression of FLAG-PML (isoform III) in mammalian cells was provided by Pier Paolo Pandolfi (Harvard Medical School, Boston, MA).

### Recombinant proteins

Recombinant His-tagged ZNF261 (amino acids 1-495) was expressed in the presence of 10 µM ZnCl_2_ in *E. coli* and purified using Ni-NTA agarose according to Manufacturer's instructions (Qiagen). For analytical ultracentrifugation experiments, MYM domains (amino acids 236–490) were expressed as His-tagged thioredoxin fusion proteins. Cultures were grown in the presence of 10 µM ZnCl_2_ at 37°C. When the OD reached ∼0.7, expression was induced with 50 µM IPTG. The temperature was adjusted to 16°C and expression was carried out overnight. His-tagged MYM domains were purified using HisTrap columns according to Manufacturer's instructions (GE Healthcare). Purified His-tagged MYM domains were incubated with His-tagged TEV protease overnight at 4°C. The protein sample was applied to a HisTrap column and untagged MYM domains were collected in the flow-through. Untagged MYM domains were separated from impurities using a MonoS column (GE Healthcare) followed by gel filtration over a Superdex 200 column (GE Healthcare). GST, GST-tagged SUMO-1, SUMO-2, SUMO-2(x3), and SUMO-2(QFI) were purified by affinity chromatography on glutathione-Sepharose 4B beads (GE Healthcare) according to manufacturer's instructions.

### Transfections

Transfections were performed in 35×10–mm tissue culture dishes with 1.0 µg of DNA and 2.5 µl of Lipofectamine 2000 (Invitrogen) per transfection or 60×10-mm tissue culture dishes with 2.5 µg of DNA and 6.25 µl of Lipofectamine 2000 (Invitrogen) per transfection. Immunofluorescence or immunoprecipitation experiments were performed 24 hours later.

### Zinc content analysis

Zinc content of ZNF261 (1-495) samples (3 µM and 0.5 µM) were determined by ICP-MS (Inductively Coupled Plasma Mass Spectrometry) using an Agilent 7500ce ICP-MS (Agilent). Samples were diluted to a final concentration of 1% HNO_3_ and a final volume of 1.5 ml. A standard calibration curve was generated from samples containing 0, 1, 5, 10, 50, 100, 500 µg/L Zn^2+^. Prior to analysis ZNF261 (amino acids 1-495) samples were dialyzed overnight against 25 mM HEPES, pH 7.3, 350 mM NaCl, 5 mM β-mercaptoethanol, 10 µM ZnCl_2_ (Untreated), or 50 mM EDTA, 100 mM sodium acetate pH 5.5, or 6 M GuHCl, or 10 mM 1,10-phenanthroline, or 10 mM 1,7-phenanthroline.

## Results

### ZNF261 and ZNF198 are SUMO-binding proteins

**Figure 1:**
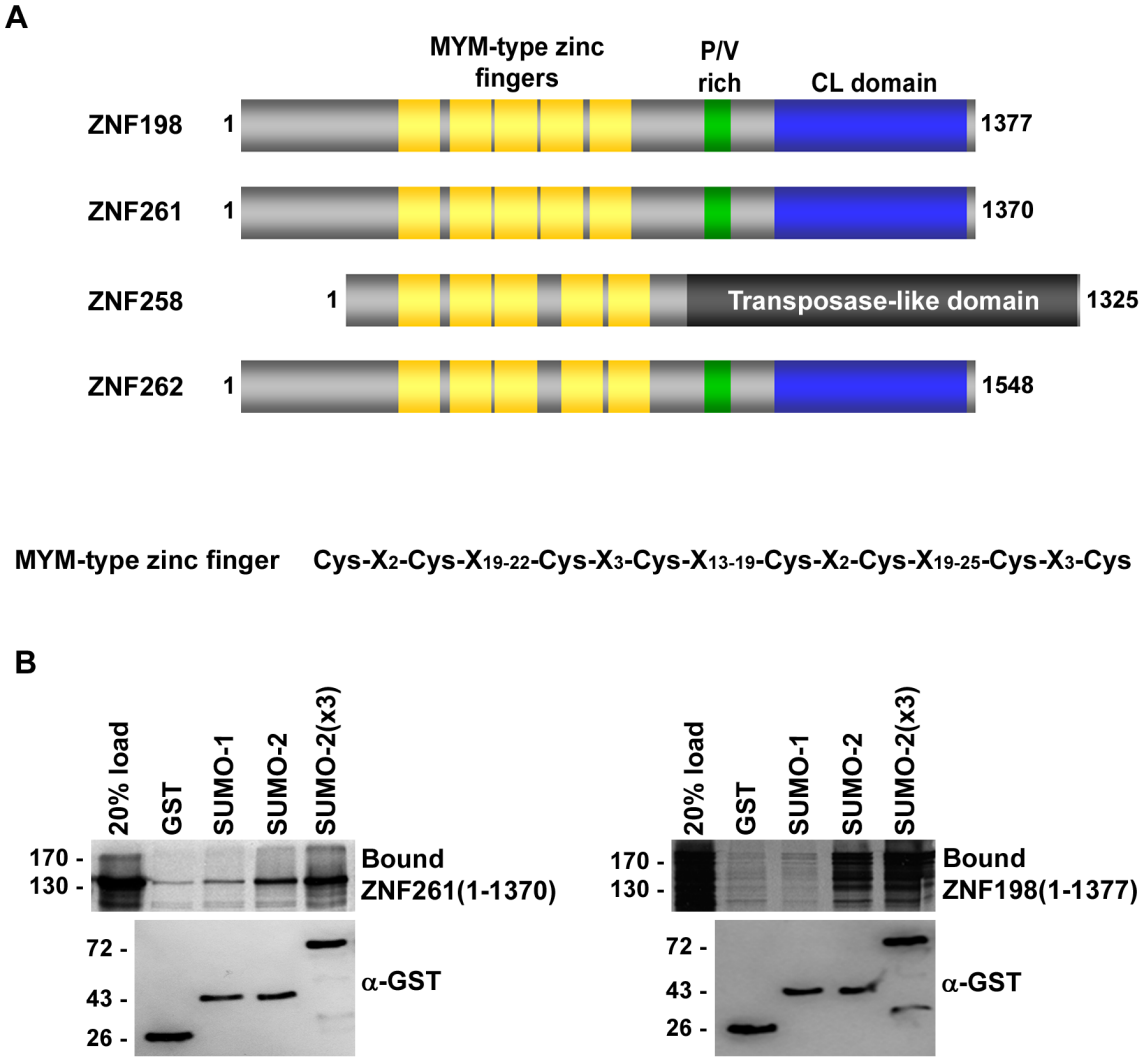
MYM-type zinc finger containing proteins ZNF261 and ZNF198 have SUMO-binding activity. A: Schematic diagrams of MYM-type zinc finger containing proteins. Amino acid number is shown next to each schematic diagram. Locations of the myeloproliferative and mental retardation-type zinc fingers (MYM-type zinc fingers, gold), proline/valine-rich domains (P/V-rich, green), and Cre-like domains (CL domain, blue) are shown. The MYM-type zinc finger consensus motif where X is any amino acid is shown below. **B**: Full-length ZNF261 or ZNF198 proteins were *in vitro* transcribed and translated in the presence of [^35^S] methionine and bound to immobilized GST-tagged SUMO-1, SUMO-2 or SUMO-2(x3). Bound proteins were eluted with SDS-sample buffer and analyzed by SDS-PAGE and autoradiography. Binding to GST alone was performed as a negative control. All binding assays contained equivalent amounts of GST and GST-tagged SUMO proteins as determined by immunoblot analysis of eluted proteins with an anti-GST antibody.

We performed SUMO-binding assays on protein microarrays containing ∼4,000 unique human proteins that are known or predicted to function as transcription factors [26]. Microarrays were probed with fluorescently labeled SUMO-1, SUMO-2, and a linear polymeric SUMO-2 referred to as SUMO-2(x3) that consists of three consecutive SUMO-2 proteins. Among the identified proteins with preferential SUMO-2 binding was ZNF261, which contains a stretch of unique tandem zinc fingers called MYM-type zinc fingers. The human genome encodes four proteins including ZNF261, ZNF198, ZNF258, and ZNF262 that contain tandem repeats of MYM-type zinc fingers (Figure 1A). Depending on the definition of the MYM-type zinc finger consensus motif, ZNF198 and ZNF261 contain either 5 or 9 MYM-type zinc fingers. The MYM-type zinc finger consensus motif has been defined as Cys-X_2_-Cys-X_19-22_-Cys-X_3_-Cys-X_13-19_-Cys-X_2_-Cys-X_19-25_-Cys-X_2_-Cys where X is any residue [12]. Thus, ZNF198 and ZNF261 contain 5 MYM-type zinc fingers. However, the MYM-type zinc finger has also been defined as Cys-X_2_-Cys-X_19-24_[F/Y]-Cys-X_3_-Cys-X_3_[F/Y] giving rise to 9 MYM-type zinc fingers in ZNF198 and ZNF261 [18].

To validate the protein microarray binding results, full-length ZNF261 was expressed in rabbit reticulocyte lysate and incubated with GST, GST-tagged SUMO-1, SUMO-2, SUMO-2(x3) immobilized on glutathione-coated plates (Figure 1B). Consistent with our protein microarray results, we found that ZNF261 interacted preferentially with SUMO-2 compared to SUMO-1. Additionally, we demonstrated that ZNF198, a second member of the MYM-type zinc finger protein family that was not found on the protein microarray, also displayed preferential binding to SUMO-2 (Figure 1B).

### The MYM-type zinc fingers in ZNF261 and ZNF198 mediate SUMO-binding

**Figure 2:**
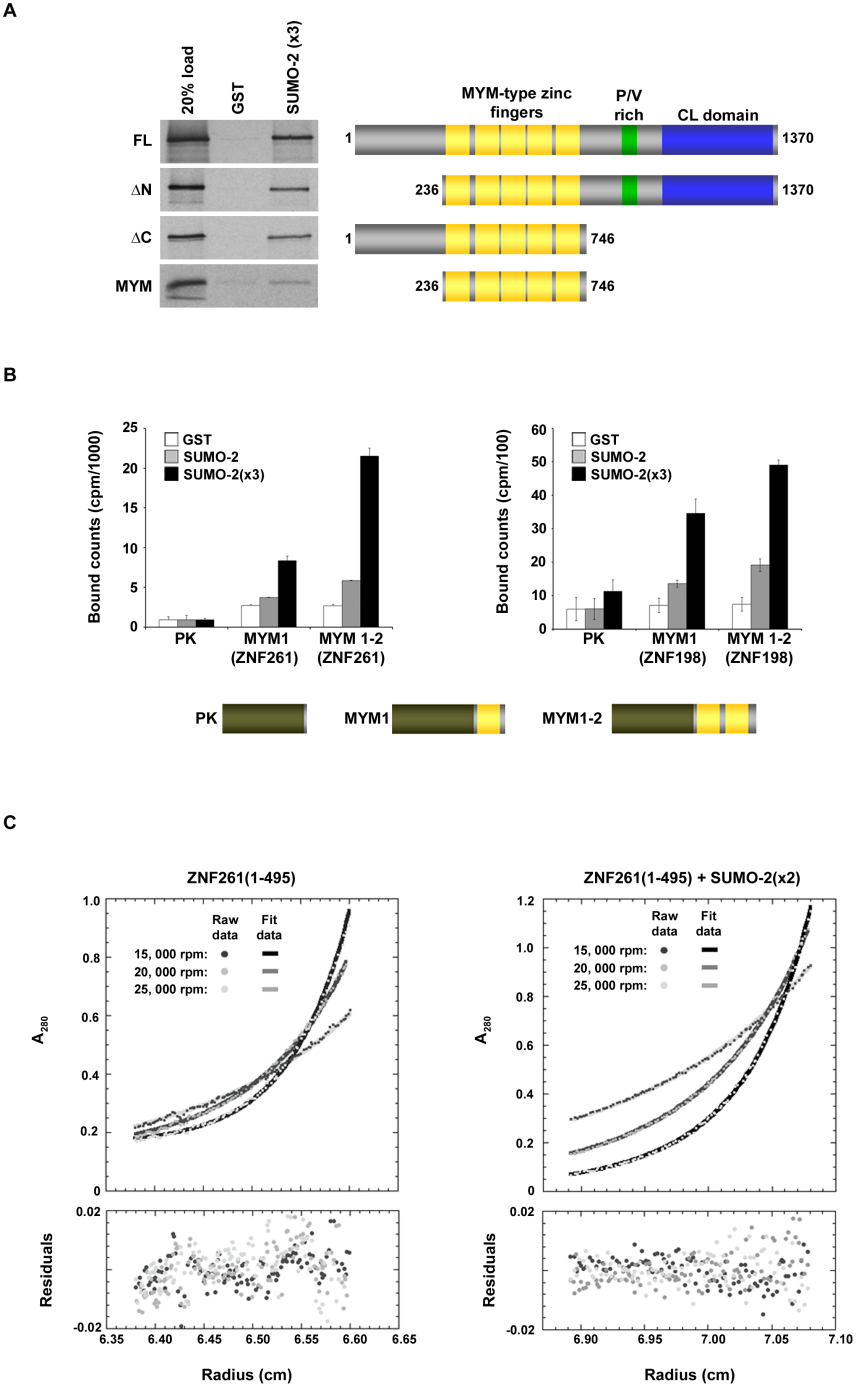
MYM-type zinc fingers in ZNF261 and ZNF198 are involved in SUMO-binding. A: GST and GST-SUMO-2(x3) were immobilized on glutathione coated plates and incubated with ^35^S-labeled full-length or N- or C-terminus truncation fragments of ZNF261. Bound proteins were eluted with SDS-sample buffer and analyzed by SDS-PAGE and autoradiography. **B**: Pyruvate kinase or pyruvate kinase fused to the MYM-type zinc fingers from ZNF261 or ZNF198 were incubated with immobilized GST (white), GST-SUMO-2 (grey), and GST-SUMO-2(x3) (black). Unbound proteins were removed by washing and bound proteins were determined by liquid scintillation detection of eluted proteins. Plotted values represent the mean +/− S.D. from three independent experiments. **C**: ZNF261 was run at three concentrations in the analytical ultracentrifuge (10, 20, and 40 µM). Representative sedimentation data from the 20 µM ZNF261(1-495) data set is shown in the left panel. The data was globally fit to a single species model and the residuals between the calculated and experimental absorbance are shown below. The residuals appear randomly scattered around zero indicating that a single species model describes the data. ZNF261(1-495) (20 µM) was run with three concentrations of SUMO-2(x2) (20, 40, and 80 µM) in the analytical ultracentrifuge. Representative sedimentation data of the 20 µM ZNF261 and 80 µM SUMO-2(x2) data set is shown in the right panel. Data were globally fit to an A+B → AB model and the residuals between the calculated and experimental absorbance are shown below. The global reduced chi-squared value was 3.13.

To further investigate and delineate the SUMO-binding activity of ZNF261, we deleted either the N- or C-terminal domains and again performed *in vitro* SUMO-binding assays (Figure 2A). Binding studies using deletion mutants demonstrated that both the N- and C-terminal domains in ZNF261 are dispensable for SUMO-binding. Moreover, the zinc-finger domain alone was sufficient for SUMO binding. Although deleting the N-terminal 235 amino acids appeared to reduce the SUMO-binding activity of the zinc-finger domain, these extreme N-terminal residues alone did not bind SUMO (Figure S1).

To determine whether single MYM-type zinc fingers are able to act as SUMO-interacting motifs we fused the first one or two zinc fingers of ZNF261 to pyruvate kinase and performed binding assays. The molecular weight of a single MYM domain is ∼9 kDa and fusion of the MYM domain to pyruvate kinase enhanced expression in our rabbit reticulocyte system. Pyruvate kinase alone had minimal affinity for SUMO-2 or SUMO-2(x3) (Figure 2B). When pyruvate kinase fused with a single MYM-type zinc finger was translated in rabbit reticulocyte and binding assays were performed we detected a 1.5-fold increase in binding to SUMO-2 and a 3-fold increase in binding to SUMO-2(x3) compared to GST alone, as assessed by quantitative comparison of bound [^35^S]-labeled protein (Figure 2B). Adding a second MYM-type zinc finger to pyruvate kinase further increased the binding to both SUMO-2 and SUMO-2(x3) by more than 2-fold, indicating that individual motifs contribute to SUMO recognition (Figure 2B). Similar results were obtained in SUMO-binding assays using the MYM-type zinc fingers of ZNF198 fused to pyruvate kinase (Figure 2B). Taken together, these results indicate that individual MYM-type zinc fingers in ZNF261 and ZNF198 are sufficient for SUMO-binding.

To further characterize interactions between the MYM-type zinc fingers of ZNF261 and SUMO-2, we determined the dissociation constant and binding stoichiometry of a ZNF261:SUMO-2 complex using equilibrium analytical ultracentrifugation. Experiments were performed with a recombinant fragment of ZNF261 containing the first two MYM-type zinc fingers (amino acids 237-490) and SUMO-2(x2), a linear fusion of two consecutive SUMO-2 proteins. Analytical equilibrium ultracentrifugation of ZNF261(1-495) alone revealed that this fragment behaves as a monomer in solution (Figure 2C, left panel). The same experiment was then performed using ZNF261 in combination with three different concentrations of SUMO-2(x2) (Figure 2C, right panel). By globally fitting the centrifugation data to an A+B → AB binding model, we determined that a 1∶1 complex formed with a dissociation constant of 56.3 µM (with a 95% confidence interval of 35.8 µM to 91.2 µM).

### MYM-Type Zinc Fingers and classical SIMs interact with a common surface on SUMO-2

**Figure 3:**
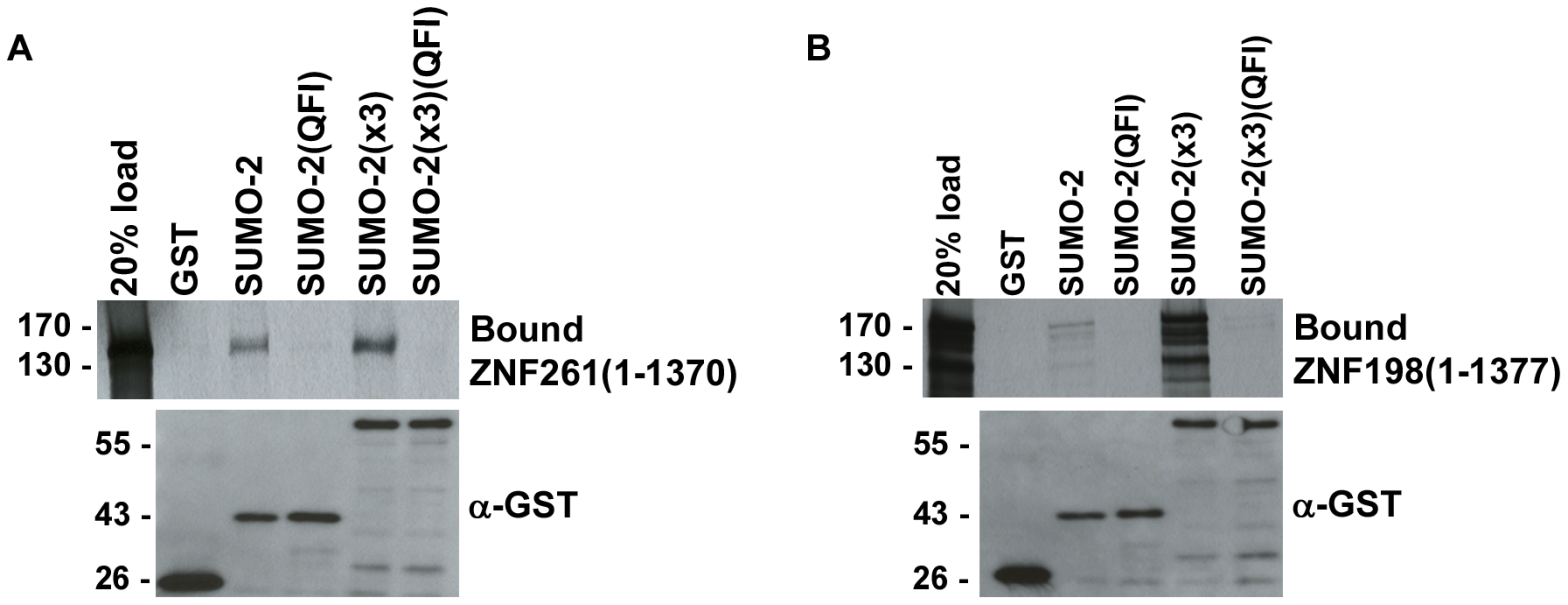
MYM-type zinc fingers interact with the α-helix and second β-strand in SUMO-2. *In vitro* expressed full-length ZNF261 and ZNF198 were incubated with GST or GST-tagged SUMO-2, SUMO-2(QFI), SUMO-2(x3), and SUMO-2(x3)(QFI). Bound proteins were eluted with SDS-sample buffer and analyzed by SDS-PAGE and autoradiography. All binding assays contained equivalent amounts of GST and GST-tagged SUMO proteins as determined by immunoblot analysis of eluted proteins with an anti-GST antibody.

The archetypal consensus SIM forms a β-strand that inserts between the α-helix and second β-strand in SUMO [4], [7]-[9], [27]. This interaction can be disrupted by alanine substitutions of Q35, F36 and I38 in the second β-strand of SUMO-2. For example, defective interactions between SUMO-2(QFI) and SIM containing proteins BLM and HIPK2 have been previously demonstrated [28]. To determine whether the MYM-type zinc finger interacts with the α-helix and second β-strand in SUMO-2, we performed binding assays using wild-type and mutant SUMO-2(QFI) proteins. We were unable to detect binding to SUMO-2(QFI) or SUMO-2(x3)(QFI) by ZNF261 or ZNF198 (Figure 3). Thus, residues in the second β-strand of SUMO-2 are equally important for interactions with classical or MYM-type zinc fingers.

### MYM-type zinc fingers in ZNF261 coordinate Zn^2+^

The MYM-type zinc finger consensus sequence is “Cys-X_2_-Cys-X_19-22_-Cys-X_3_-Cys-X_13-19_-Cys-X_2_-Cys-X_19-25_-Cys-X_3_-Cys” where X is any amino acid [12]. Most zinc finger proteins use four cysteine residues in a tetrahedral configuration to coordinate a zinc atom, however pentahedral and hexahedral coordination has also been reported [29], [30]. Thus, MYM-type zinc fingers are putative zinc-binding motifs where each motif is predicted to bind two zinc atoms with each zinc atom tetrahedrally coordinated by four cysteine residues. To investigate the zinc coordinating properties of MYM-type zinc fingers, we expressed and purified a recombinant fragment of ZNF261(1-495) containing the first two MYM-type zinc fingers and measured the zinc content by ICP-MS. Consist with tetrahedral coordination, we detected approximately 2 zinc atoms bound per MYM-type zinc finger (Table 1).

**Table 1:** ICP-MS analysis of zinc content in ZNF261(1-495).

ZNF261 (µmol/L)	Treatment	Zinc (µmol/L)	ZNF261:Zinc	∼ZNF261:Zinc
0.25	Untreated	0.95	1∶3.8	1∶4
1.50	Untreated	5.70	1∶3.8	1∶4
0.25	GuHCl	0.02	1∶0.1	1∶0
1.50	GuHCl	0.50	1∶0.3	1∶0
0.25	EDTA	0.02	1∶0.08	1∶0
1.50	EDTA	0.37	1∶0.25	1∶0
0.25	1,10-phenathroline	0.12	1∶0.5	1∶1
1.50	1,10-phenathroline	1.50	1∶1	1∶1
0.25	1,7-phenathroline	1.03	1∶4	1∶4
1.50	1,7-phenathroline	5.25	1∶3.5	1∶4

Recombinant ZNF261(1-495) was dialyzed overnight against assay buffer (25 mM HEPES pH 7.3, 350 mM NaCl, 5 mM β-mercaptoethanol) or assay buffer containing one of the following treatments: 6 M GuHCl, 50 mM EDTA, 10 mM 1,10-phenathroline, or 10 mM 1,7-phenathroline. Zinc content was determined by Inductively Coupled Plasma Mass Spectrometry (ICP-MS).

To determine whether various chelating agents effectively remove zinc from the MYM-type zinc finger, we subjected recombinant ZNF261(1-495) to several chelating treatments and measured the zinc content by ICP-MS (Table 1). As a negative control, we measured the Zn^2+^ content after denaturation with 6 M guanidine hydrochloride, a condition in which 10% of the Zn^2+^ ion remained bound relative to untreated protein. After treatment with EDTA or 1,10-phenanthroline, the percent of Zn^2+^ ion remaining bound to ZNF261(1-495) was 7% and 28% compared to untreated protein, respectively. As expected, >90% of the Zn^2+^ ion remained bound to ZNF261 after treatment with 1,7-phenanthroline, the non-chelating analog of 1,10-phenanthroline. Thus, MYM-type zinc fingers are zinc-binding motifs and zinc can be removed using standard chelating agents.

### MYM-Type Zinc Fingers bind to SUMO in the presence of zinc chelators

**Figure 4:**
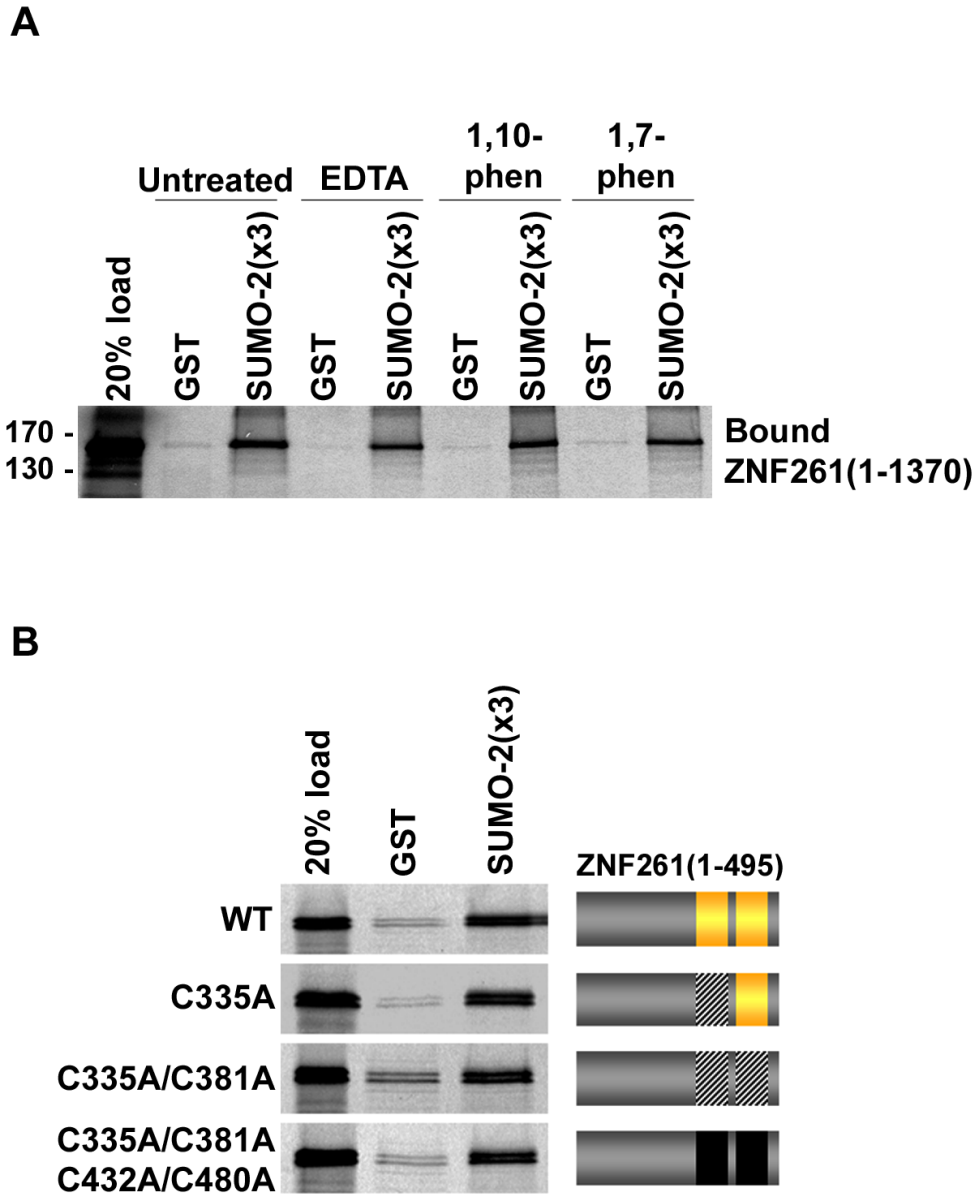
Zinc chelation does not significantly perturb SUMO-binding activity of ZNF261. A: *In vitro* expressed full-length ZNF261 proteins were incubated overnight with immobilized GST or GST-tagged SUMO-2(x3) in assay buffer (untreated) or assay buffer containing 50 mM EDTA/100 mM sodium acetate, pH 5.5 (EDTA treated), or 1,10-phenanthroline (1,10-phen), or 1,7-phenanthroline (1,7-phen). Unbound proteins were washed away and bound proteins were visualized by SDS-PAGE followed by autoradiography. **B**: Immobilized GST or GST-tagged SUMO-2(x3) was incubated with ^35^S-labeled ZNF261(1-495) wild-type or ZNF261(1-495) containing cysteine to alanine substitutions. Bound proteins were eluted with SDS-sample buffer and analyzed by SDS-PAGE and autoradiography.

To determine whether zinc coordination is necessary for the SUMO-binding activity of MYM-type zinc fingers, we performed *in vitro* binding assays in the absence and presence of metal chelators. Full-length ZNF261 was expressed in reticulocyte lysate and incubated with GST and GST-tagged SUMO-2(x3) in the absence or presence of metal chelators. Surprisingly, similar amounts of ZNF261 were pulled down by SUMO-2(x3) in standard assay conditions and in the presence of either EDTA, 1,10-phenanthroline, or 1,7-phenanthroline (Figure 4A).

Because reticulocyte lysate contains ions and proteins that could interfere with zinc chelation, we also performed binding assays using purified recombinant His-tagged ZNF261(1-495) (Figure S2A). GST and GST-tagged SUMOs were immobilized on glutathione-coated plates and then incubated with His-tagged ZNF261(1-495) that had been dialyzed against assay buffer or assay buffer containing EDTA or 1,10-phenanthroline. Consistent with our binding assays using ZNF261 produced in reticulocyte lysate, similar amounts of recombinant ZNF261(1-495) bound to SUMO in the absence and presence of zinc chelators (Figure S2B).

To further demonstrate that MYM-type zinc fingers can bind SUMO independently of zinc we performed binding assays using ZNF261(1-495) containing cysteine residues within the MYM-type zinc fingers mutated to alanine. ZNF261(1-495) contains two MYM-type zinc fingers each with 8 cysteine residues for a total of 16 cysteine residues. Wild-type and cysteine mutant ZNF261 proteins were transcribed and translated in rabbit reticulocyte lysate and binding assays were performed under standard assay conditions. Minimal differences in SUMO-binding activity were observed between wild-type and cysteine mutant ZNF261 proteins containing 1, 2, or 4 cysteine residues mutated to alanine (Figure 4B). Taken together, these results indicate that zinc is dispensable for SUMO-binding by MYM-type zinc fingers.

### Removal of zinc does not significantly perturb the secondary structure of ZNF261

We hypothesized that MYM-type zinc fingers bind to SUMO in the presence of metal chelators because the secondary structure of the MYM-type zinc finger is not significantly perturbed by zinc removal. To test this hypothesis we measured the secondary structure of untreated and EDTA-treated recombinant ZNF261(1-495) by CD spectropolarimetry. As a negative control, we completely denatured ZNF261(1-495) with 6 M GuHCl and observed that the CD spectrum showed a significant loss of ZNF261 secondary structure (Figure S2C). Similar CD spectra were obtained for untreated and EDTA-treated ZNF261(1-495) indicating that the secondary structure of ZNF261(1-495) does not undergo significant perturbation upon removal of zinc (Figure S2C).

### MYM-Type Zinc Fingers in ZNF198 are necessary but not sufficient for localization to PML-NBs

**Figure 5:**
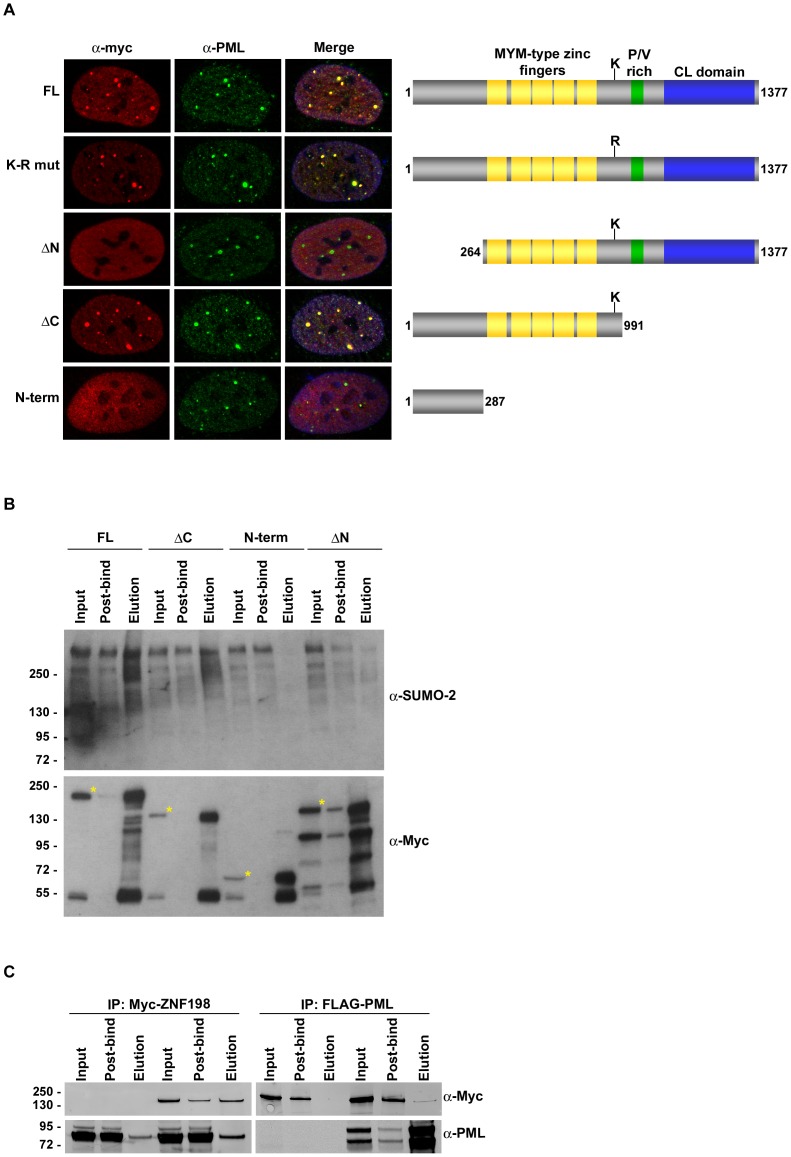
The N-terminus and MYM-type zinc fingers in ZNF198 are required for localization to PML-NBs. A: U2OS cells were transfected with constructs encoding myc-tagged ZNF198 full-length or truncation fragments. Localization of ZNF198 with PML-NBs was analyzed by fluorescence microscopy. **B**: Full-length and truncation fragments of myc-tagged ZNF198 were expressed in U2OS cells, immunopurified with anti-myc agarose beads, and detected with anti-myc and anti-SUMO-2 antibodies. Full-length protein bands are marked with an asterisk in each input lane. **C**: Myc-tagged ZNF198 and FLAG-tagged PML were co-expressed in U2OS cells, immunopurified with anti-myc agarose beads or anti-FLAG agarose beads, and detected with anti-myc and anti-PML antibodies.

A previous study reported that ZNF198 localizes to PML-NBs and that SUMO modification of ZNF198 on residue K963 is required for this localization [22]. To investigate whether MYM-type zinc fingers are also required for localization of ZNF198 to PML-NBs, we examined co-localization of ZNF198 truncation mutants with PML by immunofluorescence microscopy. We detected co-localization of PML and myc-tagged full-length ZNF198 wild-type, the SUMO modification site mutant (K-R mut, K963R), and the C-terminal truncation mutant (Figure 5A). Interestingly, we observed that truncation of the N-terminus prevented localization of ZNF198 to PML-NBs, and that the N-terminus alone was not sufficient for targeting ZNF198 to PML-NBs (Figure 5A).

To characterize interactions with SUMO-modified proteins in vivo, we immunopurified myc-tagged full-length ZNF198 and truncation mutants from transiently transfected cells. We detected high molecular mass SUMO-2 conjugates in immunopurifications of full-length ZNF198, as well as N- and C-terminal deletion mutants (Figure 5B). Notably, levels of SUMO-2 conjugates were reduced in immunopurifications of the N-terminal deletion mutant, correlating with the observed defect in localization to PML nuclear bodies. No SUMO-2 conjugates were immunopurified with the N-terminal fragment of of ZNF198 alone, indicating that it is not directly involved in SUMO binding.

To investigate whether ZNF198 interacts directly with PML in mammalian cells, we co-expressed FLAG-tagged PML and myc-tagged ZNF198 and performed reciprocal immunopurification experiments. Using anti-myc and anti-Flag agarose beads, we were able to co-purify ZNF198 and PML from mammalian cell extracts (Figure 5C). Unfortunately, attempts to co-purify PML with truncation mutants of ZNF198 were inconclusive. Taken together, our studies reveal that the N-terminus and MYM-type zinc fingers in ZNF198 are both required for localization to PML-NBs and that ZNF198 can interact with PML.

## Discussion

Here we report the identification and characterization of the SUMO-binding activity of the MYM-type zinc fingers in ZNF261 and ZNF198. Our characterization of the SUMO-binding activity of the MYM-type zinc finger in ZNF261 and ZNF198 includes the following findings: (1) MYM-type zinc fingers preferentially bind to SUMO-2, (2) the MYM-type zinc finger:SUMO-2 complex binding stoichiometry is 1∶1, (3) MYM-type zinc fingers and classical SIMs interact with a common surface on SUMO-2, (4) each MYM-type zinc finger coordinates two zinc atoms, (5) MYM-type zinc fingers bind to SUMO in the absence of zinc, (6) removal of zinc does not significantly perturb the secondary structure of the MYM-type zinc finger, and (7) MYM-type zinc fingers and the N-terminus of ZNF198 mediate localization to PML-NBs.

ZNF198 contains three predicted classic SIMs (two in the N-terminus and one in the zinc-finger domain), however, SUMO-binding assays using truncated ZNF198 fragments revealed that these predicted SIMs are not involved in binding to SUMO-2 modified HDAC1 [18]. Sequence analysis of ZNF261 did not reveal any classic SIMs [31]. Although it appears from our binding data that the zinc finger domain of ZNF261 alone does not bind SUMO as well as the full-length protein (Figure 2A), we do not believe that the N- or C-terminal domains have direct effects on SUMO binding, as their individual deletions had minimal impact. Consistent with this, we detected no interactions between the extreme N-terminus of ZNF261 and SUMO (Figure S1).

Although SUMO-binding activity has been formally mapped to the MYM-type zinc finger domains in only two proteins, and to individual MYM-type zinc fingers in only ZNF261, we propose that these zinc fingers represent a signature SIM. However, whether all MYM-type zinc fingers function individually as SIMs, and whether they all share similar affinities for different SUMO paralogs, remains to be formally tested. The four MYM-type zinc finger proteins expressed in mammals each contain 5 tandem MYM-type zinc fingers, suggesting a potential to interact preferentially with poly-SUMO chains, proteins mono-sumoylated at multiple tandem sites, or with multiple sumoylated proteins. Consistent with individual MYM-type zinc fingers functioning as SIMs to enhance binding to poly-SUMO, we detected more robust interactions between a ZNF261 fragment with two zinc-finger motifs and poly-SUMO-2 compared to a fragment with a single zinc-finger motif and monomeric SUMO-2. We measured the binding affinity of the two N-terminal MYM-type zinc fingers of ZNF261 for SUMO-2(x2) as ∼50 µM. In comparison, recent studies demonstrated affinities between ∼15–70 µM for interactions between individual tandem classic SIMs present in RNF4 and SUMO-2(x2) [32], [33]. Thus, MYM-type zinc fingers and classic SIMs appear to bind SUMO with comparable affinities. Future studies will be required to determine the biological significance of the interaction between tandem MYM-type zinc fingers with SUMO and also to determine whether the remaining two family members, ZNF262 and ZNF258 can bind SUMO.

The mechanisms used by SUMO-binding proteins to differentiate between SUMO-1 and SUMO-2 have not been completely defined. For several SUMO-binding proteins the charged residues adjacent to the consensus SIM confer preferential SUMO-1 binding [4], [11]. The classical SIM forms a β-strand that inserts into a groove on SUMO-1 and SUMO-2 [4], [5], [7]–[9], [27]. Our studies revealed that the MYM-type zinc fingers of ZNF198 and ZNF261 bind preferentially to SUMO-2 using the same surface on SUMO-2 recognized by the classical SIM. Understanding how the MYM-type zinc fingers preferentially bind to SUMO-2 could provide important insight into paralog selective binding and function.

Our ICP-MS analysis revealed 2 zinc atoms bound per MYM-type zinc finger, however, SUMO-binding activity and secondary structure were not significantly perturbed by zinc chelation. This effect may be related to the formation of stabilizing disulfide bridges between cysteine residues, as has been reported for two zinc finger proteins T4 gp32 metalloprotein and DnaJ [34], [35]. Although our observation that mutating the cysteines to alanine argues against a requirement of disulfide stabilization, further studies are required to investigate this possibility and any physiological significance.

An important consequence of SUMO modification is formation of multi-protein complexes assembled by interactions between modified proteins and proteins containing SIMs. For example, PML-NBs are formed by association of SUMO modified PML with proteins containing SIMs including PML [36], Daxx [37], TDG [38], RNF4 [39]–[41], and HIPK2 [42]. Consistent with SUMO binding being required for localization of these proteins to PML-NBs, we found that the MYM-type zinc fingers in ZNF198 were also necessary but not sufficient for localization to PML-NBs. Efficient recruitment of ZNF198 to PML-NBs required both the MYM-type zinc fingers and the N-terminus. We hypothesize that the N-terminus in ZNF198 may contain a PML-binding motif that in combination with the MYM-type zinc fingers allows efficient incorporation of ZNF198 into PML-NBs and association with SUMO-modified proteins. Consistent with this hypothesis, full-length ZNF198 and the C-terminal truncation mutant co-purified with higher levels of SUMO-2 conjugates compared to the N-terminal truncation mutant. Moreover, other studies have demonstrated that proteins can contain SIMs in tandem with binding domains that specifically recognize the SUMO modified protein. For example, the DNA helicase Srs2 contains a SIM in close proximity to a PCNA binding motif and both motifs are required for recognition of SUMO-modified PCNA [43].

In this study we revealed that the MYM-type zinc-finger domain mediates SUMO-binding. Moreover, a previous study demonstrated that the ZZ Zinc finger motif in HERC2 is a SUMO-binding motif [44], suggesting that multiple different classes of zinc fingers have the potential to recognize SUMO, ubiquitin, or other ubiquitin-like proteins. Consistent with this, affinity chromatography and yeast two-hybrid assays have identified several other types of zinc finger proteins with SUMO-binding activity [4], [45]. In addition, the second largest class of ubiquitin-binding domains are the ZnF domains consisting of UBZ, NZF, A20 ZnF, and ZnF UBP domains [46]. We anticipate that bioinformatics and biochemical analysis will continue to identify additional types of zinc-finger SIMs.

In summary, our studies have demonstrated that the MYM-type zinc fingers in ZNF261 and ZNF198 function as SIMs. MYM-type zinc finger containing proteins function in transcriptional regulation [18], [19], [47], prevention of telomeric fusions [21], [48], [49], and DNA repair [20]. In future studies, it will be important to explore how the SUMO-binding activities of these proteins influence their roles in these and other cellular processes.

## Supporting Information

Figure S1 **The N-terminus of ZNF261 does not bind SUMO.** *In vitro* expressed ZNF261(1-495) truncation fragments were incubated with GST or GST-tagged SUMO-2(x3). Bound proteins were eluted with SDS-sample buffer and analyzed by SDS-PAGE and autoradiography.
(TIF)

Figure S2 **Zinc chelation does not significantly perturb SUMO-binding activity or secondary structure of ZNF261.** A: Schematic Diagram of his-tagged ZNF261(1-495) used for SUMO-binding assays and CD spectropolarimetry. **B**: Immobilized GST or GST-tagged SUMO-2(x3) was incubated with recombinant His-tagged ZNF261(1-495) and bound proteins were visualized by SDS-PAGE followed by immunoblot analysis with anti-His antibody. Prior to incubation, ZNF261(1-495) was dialyzed overnight against assay buffer or assay buffer containing EDTA or GuHCl. **C**: CD spectra for 75 µM ZNF261(1-495) untreated (diamonds), EDTA treated (triangles), and GuHCl treated (squares) were obtained.
(TIF)
