# Supplementary figures and images for: Characterization of the SUMO-Binding Activity of the Myeloproliferative and Mental Retardation (MYM)-Type Zinc Fingers in ZNF261 and ZNF198
Source: PLoS One. 2014 Aug 18;9(8):e105271. doi: 10.1371/journal.pone.0105271 (PMC4136804; doi:10.1371/journal.pone.0105271)

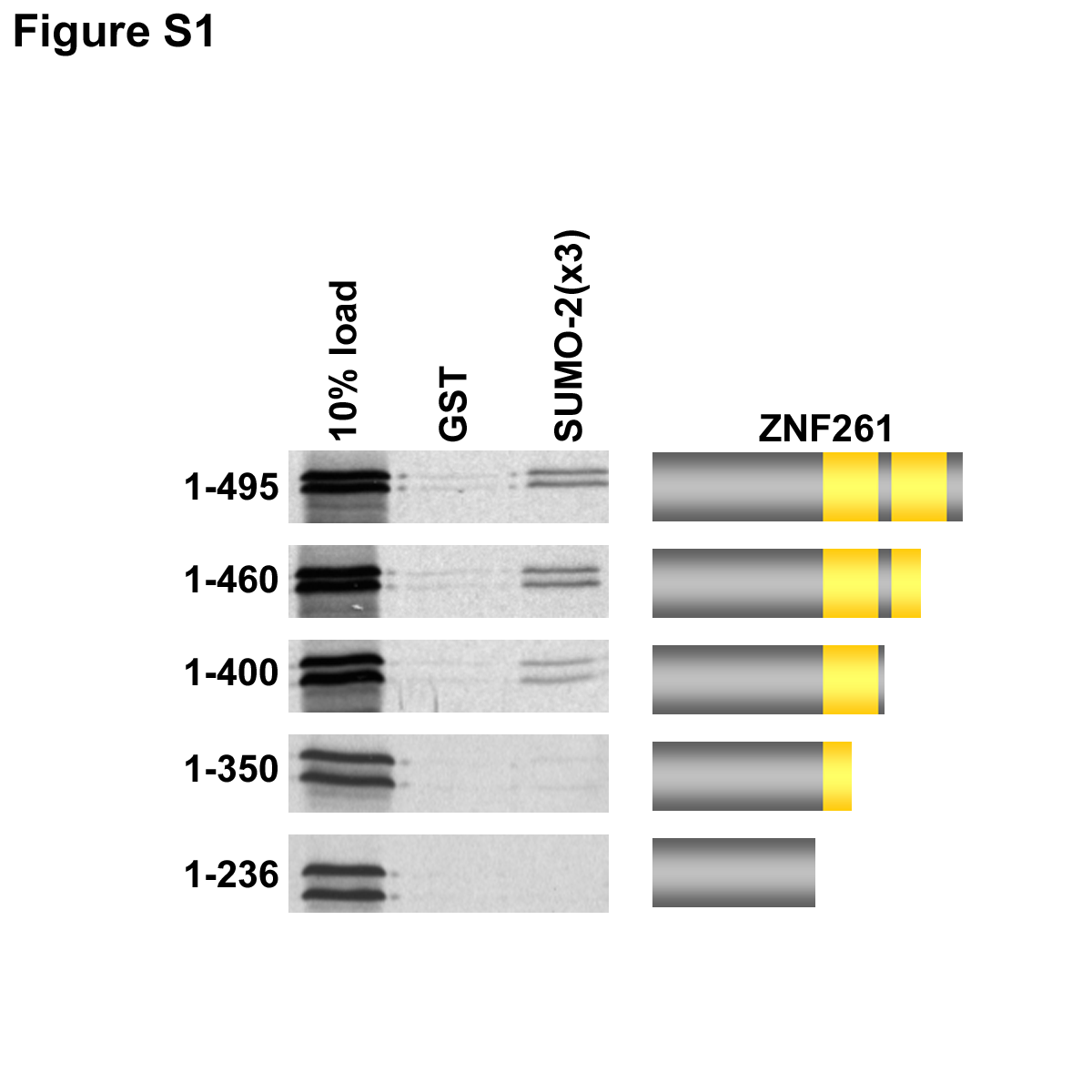

Supplement: Figure S1 — The N-terminus of ZNF261 does not bind SUMO. In vitro expressed ZNF261(1-495) truncation fragments were incubated with GST or GST-tagged SUMO-2(x3). Bound proteins were eluted with SDS-sample buffer and analyzed by SDS-PAGE and autoradiography. (TIF) [file pone.0105271.s001.tif]

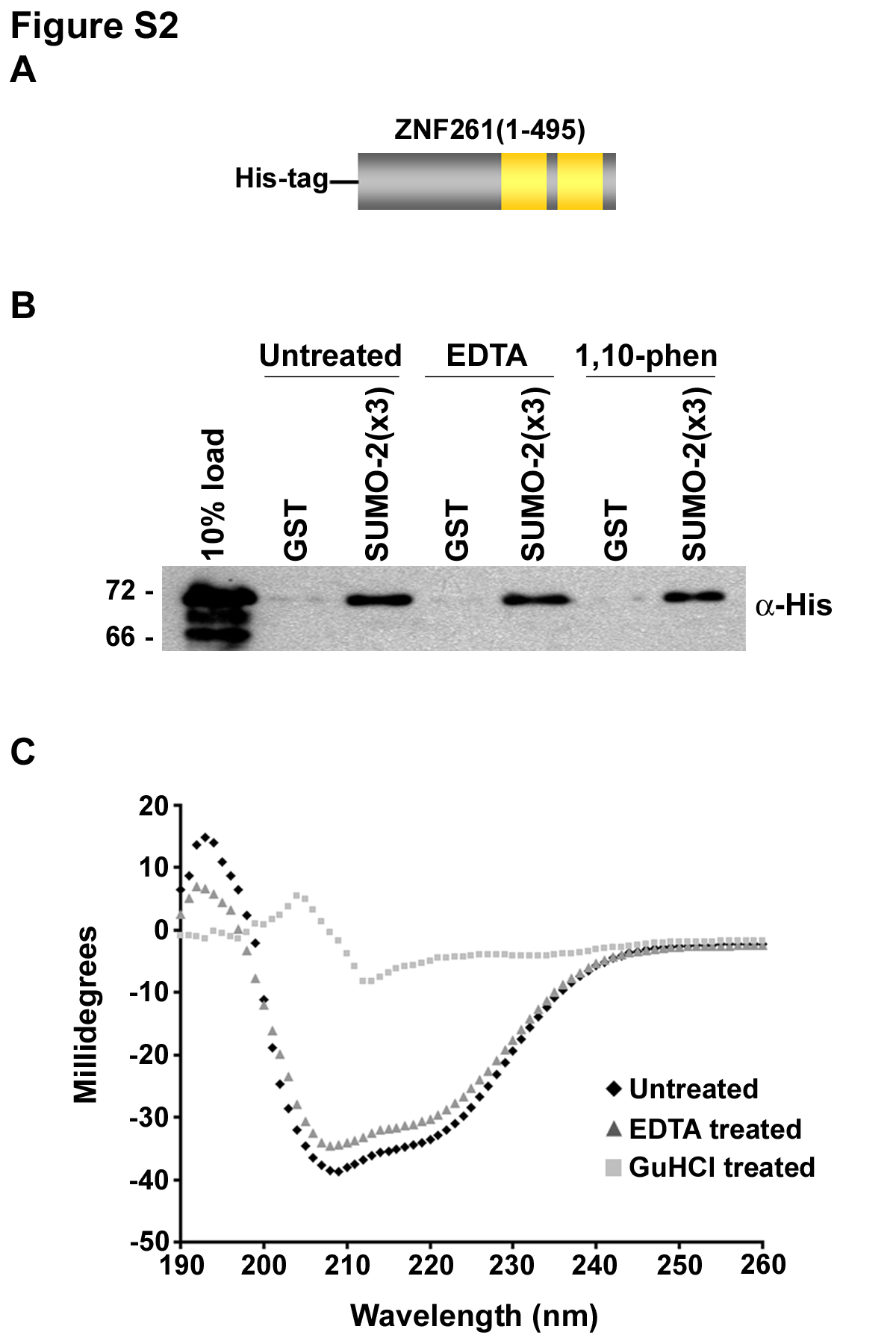

Supplement: Figure S2 — Zinc chelation does not significantly perturb SUMO-binding activity or secondary structure of ZNF261. A: Schematic Diagram of his-tagged ZNF261(1-495) used for SUMO-binding assays and CD spectropolarimetry. B: Immobilized GST or GST-tagged SUMO-2(x3) was incubated with recombinant His-tagged ZNF261(1-495) and bound proteins were visualized by SDS-PAGE followed by immunoblot analysis with anti-His antibody. Prior to incubation, ZNF261(1-495) was dialyzed overnight against assay buffer or assay buffer containing EDTA or GuHCl. C: CD spectra for 75 µM ZNF261(1-495) untreated (diamonds), EDTA treated (triangles), and GuHCl treated (squares) were obtained. (TIF) [file pone.0105271.s002.tif]
